# Selections

**Published:** 1894-06

**Authors:** 


### Selections.

### THE DANGERS OF ACONITINE.

Dr. Ferrand (*La France Médicale*, No. 47, 1893), in a discussion in the Medical Society of the Seventh Arrondissement of Paris, would have aconite rejected,—erased from general use. Even in very slight doses it is prone to give rise to accidents. He knows of a fatal case after a slight dose. Certain patients are intolerant of the drug, and cyanosis, syncope, etc., may follow very small doses. Even the tincture of aconite, in doses of ten drops, will cause toxic symptoms to appear. Intolerance is more frequently observed with aconitine than with any other drug. There is both cardiac and cerebro-spinal collapse. A case was cited where death occurred after administration of a milligramme ($\frac{1}{64}$ grain) of aconitine and five decigrammes ($7\frac{1}{2}$ grains) of antipyrin. The antipyrin seemed to augment its effects. Dr. Sottas would not like to see it thrown out of the materia medica, for he has found it of value in headaches which have resisted all other remedies; especially in a case of an old syphilitic, who suffered from terrific headache, did it do good service.—*Cincinnati Lancet-Clinic.*

---

### MERCURIC CHLORIDE.

PROBABLY no fact in connection with bacteriology has been more assiduously instilled into the minds of medical students (and pharmaceutical too, for that matter) than the surpassingly powerful antiseptic properties of solutions of corrosive sublimate. Although the disadvantages of mercuric chloride, owing to its corrosive and poisonous nature, have been generally recognized, no doubt—save, indeed, by the very heterodox—has been cherished as to the efficacy of the germicidal action of the compound.

As a matter of fact, it would seem that Koch—to whose experiments and statements the booming of sublimate was largely due—exaggerated the value of the antiseptic or overlooked the differences which must be reckoned with between laboratory experiments and clinical experience. Undoubtedly the coagulating power of mercuric chloride is practically a serious hinderance to its usefulness.

These reflections lead up to the announcement—and startling enough it proved in English medical circles—of Sir Joseph Lister, that he has entirely abandoned the use of sublimate in favor of carbolic acid. He characterizes a five-per-cent. solution as "more trustworthy as a germicide for surgical purposes than corrosive sublimate, and in other respects greatly to be preferred." A great advantage of phenol seems to be that it "has a powerful affinity for the epidermis, penetrating deeply into its substances and mixing with fatty materials in any proportion."

Looked at broadly, this expression of opinion by our most eminent apostle of antisepsis may be taken as indicative of the gradual revolution (or evolution) which is taking place in surgery. The use of antiseptics has evidently reached its zenith, and now reaction is setting in towards asepsis, the aim of which is rather to prevent the access of pathogenic micro-organisms than to attempt to destroy them *in situ.*—*London Letter*, *Bulletin of Pharmacy.*

